# Utilization frequency and patient-reported effectiveness of symptomatic therapies in post-COVID syndrome

**DOI:** 10.1186/s12889-024-19951-3

**Published:** 2024-09-23

**Authors:** Miriam Reuner, Johannes Krehbiel, Jürgen Rech, Brigitte Greiner, Isabel Schäfer, Regina Herold, Eva Morawa, Yesim Erim

**Affiliations:** 1 Department of Psychosomatic Medicine and Psychotherapy, University Hospital of Erlangen, Friedrich-Alexander University Erlangen-Nürnberg (FAU), 91054 Erlangen, Germany; 2 Post-COVID Center, University Hospital of Erlangen, 91054 Erlangen, Germany; 3 Department of Internal Medicine III, Rheumatology and Immunology, University Hospital of Erlangen, Friedrich- Alexander University Erlangen-Nürnberg (FAU), 91054 Erlangen, Germany

**Keywords:** COVID-19, Post-COVID-19, Treatment, Medication, Effectiveness

## Abstract

**Background:**

To date there is no causal treatment for post-COVID syndrome, leaving symptomatic treatments as the primary recourse. However, the practical implementation and effectiveness of these interventions remain underexplored. This study aimed to investigate the utilization frequency of symptomatic therapies and patient-reported effectiveness across various treatment modalities at a German post-COVID center.

**Methods:**

As the baseline investigation we conducted a single-cohort retrospective study to analyze the frequency of symptomatic therapies among post-COVID patients who attended the post-COVID center of the University Hospital of Erlangen, between December 2022 to July 2023. Additionally, we administered a follow-up at least 3 months after the initial presentation, using a questionnaire to assess patient-reported improvements in post-COVID symptoms associated with the symptomatic therapies received.

**Results:**

Our study included 200 patients (mean age: 44.6 ± 12.6 years; 69.0% women; mean duration since acute infection: 15.3 ± 8.3 months). Pharmacotherapy was the predominant symptomatic treatment (79.5%), with psychotropic drugs (32.5%) and analgesics (31.5%) being the most frequently prescribed. Over half of the patients (55.5%) utilized vitamins and nutritional supplements. Hospital admission rates to acute care occurred in 35.5% of cases; 33.0% underwent inpatient rehabilitation and 31.0% pursued outpatient psychotherapy. Cardiologists (76.5%), pulmonologists (67.5%), and neurologists (65.5%) were the most consulted specialists. Therapies involving medical devices were infrequently employed (12.0%). In a follow-up questionnaire (response rate: 82.5%, 6.3 ± 2.2 months post-baseline), beta-blockers were the most effective pharmacological intervention with 31.5% of patients reporting strong to very strong symptom improvement, followed by antibiotics (29.6%). Furthermore, 33.0% of the patients perceived plasmapheresis to strongly alleviate symptoms. Only a small proportion of the sample attributed a strong or very strong symptom improvement to outpatient psychotherapy (11.0%).

**Conclusion:**

This study provides initial insights into symptomatic therapy utilization and patient-reported symptom improvement in post-COVID syndrome. Further research into symptoms clusters and interdisciplinary collaboration are warranted to comprehensively address the multifaceted physical and psychological symptomatology.

**Trial registration:**

The study was registered at the German Clinical Trials Register (DRKS-ID: DRKS00033621) on March 20, 2024.

**Supplementary Information:**

The online version contains supplementary material available at 10.1186/s12889-024-19951-3.

## Introduction

As of March 2024, over 774 million COVID-19 cases have been reported to the WHO [1]. Between 1.2% and 4.8% of COVID-19 patients experience symptoms persisting for at least 12 weeks after the acute infection, significantly impacting their daily lives [2]. These enduring symptoms are collectively referred to under the diagnosis of post-COVID syndrome. The WHO defines the condition as “the continuation or development of new symptoms 3 months after the initial SARS-CoV-2 infection, with these symptoms lasting for at least 2 months with no other explanation” [3]. While fatigue, dyspnea and cognitive impairment are typical manifestations of post-COVID, more than 200 different symptoms have been reported, impacting multiple organ systems, and affecting various aspects of daily functioning [4–6].

Intensive research is currently underway to delineate the syndrome and enhance our comprehension of its pathophysiology and long-term implications. According to current knowledge, there are a multitude of factors contributing to the expansive range of symptoms observed [7, 8]. The most important are endothelial dysfunction as consequence of microvascular inflammation [9, 10], occult viral persistence [11], immune system dysregulation [12], as well as autoimmunity [13].

As of now, no singular treatment modality has been definitively established and validated for effectiveness. Current treatment strategies adopt an interdisciplinary approach to comprehensively address the variety of symptoms and complications encountered by patients [14]. The quest for an effective causal therapy for post-COVID syndrome is a central focus of numerous experimental studies worldwide. Various approaches, often adapted from treatment protocols for similar conditions, are under examination to assess their impact on post-COVID symptomatology [15, 16]. In this context, both pharmacological and non-pharmacological therapies are being explored.

Current investigations into pharmacological therapies are yielding preliminary results suggesting effectiveness. Selective serotonin reuptake inhibitors (SSRIs), for instance, show improvement in overall well-being, brain fog, sensory overload, alongside reductions in fatigue and dysautonomia [17]. Low-dose naltrexone has been shown to alleviate daily life constraints, insomnia, concentration difficulties, pain perception, and enhance energy levels [18]. Increased heart rate variability, tachycardia, and episodes of palpitations following COVID-19 infection can be significantly reduced by ivabradine or beta-blockers [19, 20]. Nevertheless, the available data is limited by small sample size and the absence of a control group, prompting a current emphasis on additional drug trials in research.

Recent studies suggest that vitamins and dietary supplements can also contribute to alleviating post-COVID symptoms. Commonly cited supplements include vitamins B, C, and D [21], along with probiotics [22] and omega-3 fatty acids [23, 24]. For instance, vitamin C supplementation has shown significant reductions in fatigue and improvements in concentration, sleep quality, and depression [25, 26], while coenzyme Q10, despite initial assumptions, does not demonstrate superiority over a placebo [27].

As blood clotting, diminished microcirculation and autoantibodies are suspected to contribute to the severity of COVID-19 [28, 29] and the onset of post-COVID symptoms [13], plasmapheresis procedures are being explored as a potential therapeutic approach. However, to date, there is no rational scientific basis for utilizing plasmapheresis to remove amyloid-fibrinogen particles (‘microclots’) in post-COVID patients [30]. Further instrumental procedures such as hyperbaric oxygen therapy, however, indicate an enhancement in physical and emotional well-being of post-COVID patients [31, 32].

Additionally, psychotherapeutic interventions exhibit promising efficacy outcomes. Cognitive-behavioral treatment for example has demonstrated improvements in subjective fatigue and disease coping [33, 34].

Rehabilitation has also emerged as an effective therapeutic approach for addressing post-COVID symptoms [35]. Current evidence indicates that rehabilitation improves various symptom clusters associated with post-COVID syndrome, including breathlessness, fatigue, anxiety, muscle strength and quality of life [36, 37]. Notably, rehabilitation appears to be an effective treatment tool regardless of whether it is conducted in a home-based or inpatient setting [37, 38]. In the current guideline from March 2023, the Association of the Scientific Medical Societies in Germany (AWMF) recommends pneumological, cardiological, neurological or psychotherapeutic rehabilitation, depending on the predominant impairment [39].

As outlined, numerous experimental studies and approaches are currently underway to explore additional and enhanced treatment options for post-COVID syndrome and its repercussions. However, there remains little data regarding the practical implementation of these interventions by patients and healthcare providers, as well as the patients’ perceptions regarding their effectiveness. In the present study conducted at a German post-COVID center, we therefore aim to investigate the frequency of therapy utilization and provide patients perspectives on the effectiveness of different treatment modalities.

## Method

### Statement of ethics and declarations

This research adheres to the principles outlined in the Declaration of Helsinki and has received approval from the Ethics Committee of the Medical Faculty at Friedrich-Alexander University Erlangen-Nürnberg (FAU), with approval numbers 22-443-B and 22–443_3-B for the retrospective baseline study and follow-up study, respectively. The study was registered at the German Clinical Trials Register (DRKS-ID: DRKS00033621) on March 20, 2024. All participants provided written informed consent prior to their participation in the study. The consent form, signed by all participants, explicitly outlined that the data would be published.

### Data collection

The study was conducted at the post-COVID center a department of the University Hospital of Erlangen, specializing in providing multidisciplinary outpatient care for individuals afflicted with post-COVID syndrome, particularly those encountering its severe presentations. At our ambulatory center, patients undergo interdisciplinary diagnostics encompassing an anamnesis interview, measurement of several issues of physical and mental symptoms and life events by an online survey including validated self-report questionnaires, laboratory assessments for inflammation markers and autoantibodies, regular consultation with internal medicine specialists, Optical Coherence Tomography Angiography (OCTA) measurements and expert evaluation of neurocognitive functions.

Our study employs a combined design. The baseline investigation, conducted at the time of the patients’ first admission, is a single-cohort retrospective study involving a thorough review of medical records including hospital discharge letters, medical reports, referrals, and rehabilitation reports as well as documented consultations and initial medical evaluations. This enabled us to capture the frequency of utilization of symptomatic therapies from 3 months post-acute COVID-19 infection until presentation at the post-COVID center. Additionally, socio-demographic, and health-related data, including post-COVID symptomatology and mental health impairments such as depression, anxiety, and somatization, were collected through an online questionnaire completed by patients at home. The results for these questionaries will be analyzed in future publications.

Moreover, we conducted a follow-up survey involving the same cohort of patients. This follow-up, administered via an online questionnaire, was carried out at least 3 months after the patients initial presentation at the post-COVID center (Supplement 1). The primary focus of this survey was to elicit the patients’ personal evaluations regarding the effectiveness of the treatment they had received thus far.

### Participants

Our study included patients aged 18 years or older attending the post-COVID center with symptoms persisting for at least three months following SARS-CoV-2 infection, as confirmed by their general practitioner. Exclusion criteria encompassed health impairments attributable to COVID-19 vaccination. Specifically, we excluded individuals who experienced symptom onset within one month of vaccination and linked these symptoms directly to the COVID-19 vaccine. This criterion ensured that the study concentrated solely on post-COVID syndrome, eliminating potential vaccine-related confounders.

### Sociodemographic variables

The following variables were evaluated: sex, age, level of education, marital and parental status, employment status.

### Pre-existing mental and physical health conditions

Pre-existing conditions were identified through examination of medical records and patient history obtained during their visit to the post-COVID center. All documented diagnoses were confirmed to precede the acute COVID-19 infection, ensuring clear differentiation from symptoms emerging post-COVID.

### COVID-19-related variables

The time between the acute SARS-CoV-2 infection and the patient’s referral to the post-COVID center was calculated to indicate the duration of post-COVID symptoms and the period available for seeking treatment. Prior to presentation in our facility general practitioners supplied details regarding the course of the acute SARS-CoV-2 infection, which was categorized into four groups: (1) asymptomatic; (2) symptomatic, managed at home or on an outpatient basis; (3) symptomatic, requiring inpatient therapy without intensive care admission; and (4) symptomatic, necessitating inpatient therapy with intensive care admission. Additionally, internal ICD-10 diagnoses were extracted from the medical reports of the post-COVID center.

### Paraclinical findings

At our post-COVID center, transthoracic echocardiograms (TTEs) conducted at least three months post-acute COVID-19 infection by either a general practitioner or an outpatient cardiologist were a prerequisite for admission. This requirement was established due to the risk of persistent cardiac complications such as myocarditis, pericarditis, myocardial infarction, and dysrhythmias following acute COVID-19 infection [14].

In our study, we evaluated these TTEs using predefined criteria. Our assessment focused on key indicators of cardiac insufficiency related to COVID-19, including ejection fraction, pulmonary arterial pressure, and the presence of a dilated left atrium or pericardial effusion. This evaluation was critical for tailoring patient care and determining the prevalence of cardiac abnormalities in our post-COVID cohort, influencing treatment decisions.

### Pharmacotherapy

The medication intake was assessed specifically including medications newly prescribed for post-COVID syndrome, initiated at least three months after the acute COVID-19 infection. To categorize the medications, a classification system based on the ATC index [40] was employed, resulting in 14 distinct classes: Psychotropic drugs, antiepileptic drugs, glucocorticoids, inhaled antiasthmatic agents, analgesics, anticoagulants, cardiac medications, antihypertensives, lipid-lowering agents, antibiotics, antivirals, antihistamines, biologics, and other medications.

### Intake of vitamins and dietary supplements

Information regarding the consumption of vitamins and dietary supplements was derived from either patient self-reports or medical records. Only dietary supplements prescribed anew or self-administered following the onset of post-COVID symptoms were included.

### Inpatient treatment

In this study, inpatient care was divided into two categories. The first category encompassed acute inpatient treatment, which aimed at providing immediate care to either cure or substantially alleviate the post-COVID symptoms. The second category focused on inpatient rehabilitation, targeting the consequences of the condition with the objective of reinstating employability. Data for this assessment stems from self-reported information obtained during the initial medical history interview with the patients at our facility, supplemented by a comprehensive review of their medical records.

### Outpatient referrals and treatment

Outpatient referrals to various specialists such as cardiologists, pulmonologists, psychiatrists, neurologists, and other relevant healthcare providers were assessed. The initiation of outpatient psychotherapy following the onset of post-COVID symptoms was also noted. Furthermore, supplementary therapies including physiotherapy, ergotherapy, and speech therapy were recorded.

### Instrumental procedures

The term ‘instrumental procedures’ refers to therapeutic methods conducted with the use of medical devices. Our research centered on several apheresis techniques including plasmapheresis, immunoadsorption, and H.E.L.P. (heparin-induced extracorporeal LDL precipitation) apheresis. Furthermore, data concerning the utilization of hyperbaric oxygenation, IHHT (Intermittent Hypoxic Hyperoxic Training), cryotherapy, as well as hyperthermia treatment were collected.

### Subjectively perceived effectiveness of the therapeutic intervention

As part of our follow-up survey, we administered an online questionnaire to gather patient perspectives on the effectiveness of various therapies in managing post-COVID symptoms (Supplement 1).

Invitations to participate in the survey were initially sent via email, with formal letters sent by mail to alleviate importance. Subsequent email reminders were sent during the 1st and 2nd weeks post-invitation. After three weeks, patients were contacted by phone, followed by a final digital reminder in the 4th week.

In the questionnaire participants were prompted to identify therapies they had undergone to alleviate their post-COVID symptoms from predefined categories (medication, instrumental procedures, psychological treatment, rehabilitation, outpatient treatment, therapeutic movement, relaxation methods and supplementary medicine). Additionally, they were provided with a free-text field to voluntarily specify any additional therapies not listed.

Participants were then asked to evaluate the effectiveness of these therapies in alleviating their post-COVID symptoms, categorizing improvements into four levels: 0 = no improvement, 1 = slight improvement, 2 = strong improvement, and 3 = very strong improvement.

### Data analysis

To provide a comprehensive overview of the research cohort, descriptive statistics including absolute and relative frequencies, mean values and standard deviations were calculated to delineate sociodemographic factors, COVID-related characteristics, pre-existing mental and physical health conditions, and therapy utilization. Statistical analyses were performed using SPSS Version 28 (IBM Corporation, Armonk, New York).

## Results

### Recruitment

A total of 205 patients were screened for the study, all referred to our facility between December 2022 and July 2023. To this end, we employed a consecutive sampling method by including patients in the order they presented at our post-COVID center. Five patients were excluded for analysis as they sought care at the post-COVID center due to enduring symptoms related to vaccination rather than post-COVID symptoms. Therefore, a total of *N* = 200 patients were included for analysis.

### Sociodemographic variables

The sociodemographic profile of our research cohort is presented in Table 1. The majority of participants were female (69.0%, *n* = 138), with an average age of 44.6 years (SD = 12.6). Notably, the 40–59 age group was most prevalent, representing 54% (*n* = 108) of the sample.

**Table 1:** Socio-demographic data of the research cohort

Variables	Total sample (*N* = 200)
**Sex, n (%)**	
Women	138 (69.0)
Men	62 (31.0)
**Age**,** years**	
M (SD)	44.6 (12.6)
Range	19–79
**Age group, n (%)**	
18–29	28 (14.0)
30–39	39 (19.5)
40–49	57 (28.5)
50–59	51 (25.5)
≥ 60	25 (12.5)
**Education level, n (%)**	
Without certificate	1 (0.5)
Secondary school	91 (45.5)
High school certificate	23 (11.5)
University certificate	71 (35.5)
Doctoral degree	4 (2.0)
Missing	10 (5.0)
**Employment status, n (%)**	
Full-time employed	40 (20.0)
Part-time employed	42 (21.0)
Sick on leave/ unable to work	81 (40.5)
Unemployed	9 (4.5)
Retired/ pensioned	5 (2.5)
Others	13 (6.5)
Missing	10 (5.0)
**Marital status, n (%)**	
Single without partnership	37 (18.5)
Single with partnership	33 (16.5)
Married	108 (54.0)
Divorced/ In separation	10 (5.0)
Widowed	2 (1.0)
Missing	10 (5.0)
**Has children, n (%)**	
Yes	113 (56.5)
No	77 (38.5)
Missing	10 (5.0)

The education level was notably high, with 37.5% (*n* = 75) of participants holding a university degree, and 11.5% (*n* = 23) possessing a high school diploma. Regarding marital status, 54% (*n* = 108) were married, and 56.5% (*n* = 113) reported having children. Additionally, 20% (*n* = 40) were engaged in full-time employment. A significant portion of the cohort, 40.5% (*n* = 81), was on sick leave due to persistent post-COVID symptoms.

### Preexisting mental and physical health conditions

The prevalence of preexisting mental and physical health conditions within our cohort is detailed in Table 2. Among the 200 individuals studied, 36.5% (*n* = 73) had at least one preexisting mental illness prior to contracting COVID-19. Clinical depression was the most prevalent, affecting 21.5% (*n* = 43) of participants, followed by somatoform disorders (9.5%, *n* = 19) and PTSD (5.0%, *n* = 10).

**Table 2:** Pre-existing mental and physical illnesses of the study sample

Disease groups	Total sample (*N* = 200)
**Cardiovascular disease, n (%)** ^*^	
Arterial hypertension	38 (19.0)
Coronary artery disease (CAD)	6 (3.0)
S/P pulmonary embolism (PE) / deep vein thrombosis (DVT)	4 (2.0)
S/P myocarditis	3 (1.5)
S/P myocardial infarction	3 (1.5)
Others	16 (8.0)
**Pulmonary disease, n (%)** ^*^	
Bronchial asthma	35 (17.5)
Obstructive Sleep Apnea Syndrome (OSAS)	14 (7.0)
Chronic Obstructive Pulmonary Disease (COPD)	7 (3.5)
Others	5 (2.5)
**Neurological disease, n (%)** ^*^	
Migraine with or without aura	23 (11.5)
S/P disc herniation	16 (8.0)
S/P transient ischemic attack (TIA) or stroke	5 (2.5)
Episodic or chronic tension headache	5 (2.5)
S/P traumatic brain injury (TBI)	4 (2.0)
Restless Legs Syndrome	3 (1.5)
Others	15 (7.5)
**Metabolic disorder, n (%)** ^*^	
Obesity	44 (22.0)
Hypothyroidism	21 (10.5)
Hashimoto thyroiditis	19 (9.5)
Diabetes mellitus type 2	4 (2.0)
Others	8 (4.0)
**Dermatological disease, n (%)** ^*^	
Atopic dermatitis	10 (5.0)
Psoriasis	3 (1.5)
Others	2 (1.0)
**Gastrointestinal disease, n (%)** ^*^	
Gastroesophageal Reflux Disease (GERD)	9 (4.5)
Chronic gastritis	5 (2.5)
Inflammatory bowel disease (IBD)	3 (1.5)
Celiac disease	3 (1.5)
Others	5 (2.5)
**Tissue disorders, n (%)** ^*^	
Arthrosis	10 (5.0)
Fibromyalgia	7 (3.5)
Rheumatoid arthritis	5 (2.5)
Ehlers-Danlos syndrome	2 (1.0)
Others	9 (4.5)
**Oncological diseases, n (%)** ^*^	
S/P mamma carcinoma	3 (1.5)
S/P melanoma	2 (1.0)
S/P papillary thyroid carcinoma	2 (1.0)
S/P colorectal carcinoma	2 (1.0)
Others	2 (1.0)
**Further somatic diseases, n (%)** ^*^	
Allergic rhinitis	22 (11.0)
Tinnitus	9 (4.5)
S/P infectious mononucleosis in the last 10 years	6 (3.0)
Endometriosis	4 (2.0)
Chronic kidney disease (CKD)	3 (1.5)
Others	14 (7.0)
**Mental illness, n (%)** ^*^	
Unipolar depression	43 (21.5)
Somatoform disorders	19 (9.5)
Post traumatic stress disorder (PTSD)	10 (5.0)
Generalized anxiety disorder (GAD)	8 (4.0)
Eating disorders (Anorexia, Bulimia)	5 (2.5)
Agoraphobia with panic disorder	3 (1.5)
Attention Deficit Hyperactivity Disorder (ADHD)	3 (1.5)
Others	13 (6.5)

^*^A simultaneous occurrence of multiple diagnoses was possible

Over 50 different preexisting physical conditions were identified, with obesity (22%, *n* = 44) and arterial hypertension (19.0%, *n* = 38) being the most common. Of note is the elevated prevalence of bronchial asthma, affecting 17.5% (*n* = 35) of our cohort.

### COVID-19-related variables

The average duration since SARS-CoV-2 infection was 15.3 months (SD = 8.3) as shown in Table 3. Most patients (84.5%, *n* = 169) indicated experiencing symptomatic manifestations during the acute infection, necessitating home care or outpatient treatment. Only a small minority required inpatient treatment (10.5%, *n* = 21). 32.0% (*n* = 64) of our cohort received a diagnosis of chronic fatigue syndrome (ICD-10 G93.3) based on the criteria of the Institute of Medicine (IOM) [41]. 6.0% (*n* = 12) have been experiencing the symptoms of Postural Orthostatic Tachycardia Syndrome (POTS, ICD-10 I95.1) since their COVID-19 infection.

**Table 3:** COVID-related data and paraclinical findings

Variables	Total sample (*N* = 200)
**Time since the SARS-CoV-2 infection, months**	
M (SD)	15.3 (8.3)
Range	3–41
**Course of the acute SARS-CoV-2 infection, n (%)**	
Asymptomatic	6 (3.0)
Symptomatic, self-treatment or outpatient therapy	169 (84.5)
Symptomatic, inpatient therapy without intensive care admission	14 (7.0)
Symptomatic, inpatient therapy with intensive care admission	7 (3.5)
Missing	4 (2.0)
**Somatic diagnoses first received at the post-COVID center, n (%)** ^*^	
Chronic Fatigue Syndrome (ICD-10 G93.3)^#^	64 (32.0)
Postural Orthostatic Tachycardia Syndrome (ICD-10 I95.1)	12 (6.0)
(Peri-)Myocarditis (ICD-10 I31.9, I40)	6 (3.0)
Cachexia (ICD-10 R64)	6 (3.0)
Joint inflammation (ICD-10 M06.89, M46.9)	3 (1.5)
Others	19 (9.5)
**Transthoracic echocardiography results, n (%)** ^*^	
Physiological result	157 (78.5)
Pericardial effusion	8 (4.0)
Dilatation of the left atrium	6 (3.0)
Elevated pulmonary arterial pressure	4 (2.0)
Reduced ejection fraction	3 (1.5)
Missing	24 (12.0)

^*^A simultaneous occurrence of multiple diagnoses/ results was possible

^#^Based on the criteria of the Institute of Medicine (IOM, 2015)

### Paraclinical findings

Upon presentation at the post-COVID center, 88.0% (*n* = 176) of patients had undergone transthoracic echocardiography (TTE), typically performed approximately 9.1 months (SD = 8.4) after the acute infection by either the primary care physician or cardiologist. Among these patients, 89.2% (*n* = 157) exhibited normal findings, while 10.8% (*n* = 19) showed at least one cardiac pathology, further detailed in Table 3.

Pharmacotherapy was the predominant therapeutic approach for post-COVID symptoms within our cohort, with 79.5% (*n* = 159) of patients receiving some form of medication. With 32.5% (*n* = 65) psychotropic medications were the most frequently prescribed, followed closely by analgesics at 31.5% (*n* = 63) and inhaled antiasthmatic medications at 29% (*n* = 58). Cardiac medications and glucocorticoids were each used in 21.5% (*n* = 43) of patients.

Among psychotropic drugs, mirtazapine was prescribed to 12.5% (*n* = 25) of patients, and SSRIs to 12.0% (*n* = 24). Additionally, duloxetine was given to 7% (*n* = 14) of patients, and amitriptyline to 3.5% (*n* = 7). Antiepileptic drugs, mainly pregabalin, were administered to 9% (*n* = 18) of patients.

For pain management, NSAIDs were the most frequently used analgesics, prescribed to 20.5% (*n* = 41) of patients, followed by other non-opioid analgesics at 17% (*n* = 34). Opioids, primarily tramadol and tilidine, were used in 6.5% (*n* = 13) of cases.

Cardiac medications, including beta-blockers and ivabradine, were prescribed in 18.5% (*n* = 37) of patients. Glucocorticoids, with the primary agent prednisolone, were administered to an equal percentage (18.5%, *n* = 37) as part of high-dose pulse therapy. Additional medications, such as antihypertensives, antibiotics, and antivirals, were utilized more sparingly for symptomatic relief.

### Intake of vitamins and dietary supplements

Over half of the patients, 55.5% (*n* = 111), incorporated vitamins or dietary supplements into their regimen, either upon physicians’ recommendation or independently. Among the vitamins, vitamin D was the most consumed at 28.5% (*n* = 57), followed by vitamin B at 24.5% (*n* = 49) and vitamin C at 17.5% (*n* = 35).

Regarding dietary supplements, magnesium was the most prevalent at 18.5% (*n* = 37), followed by zinc at 10.0% (*n* = 20), coenzyme Q10 at 9.0% (*n* = 18), and probiotics at 8.0% (*n* = 16).

### Inpatient treatment

**Fig. 1:**
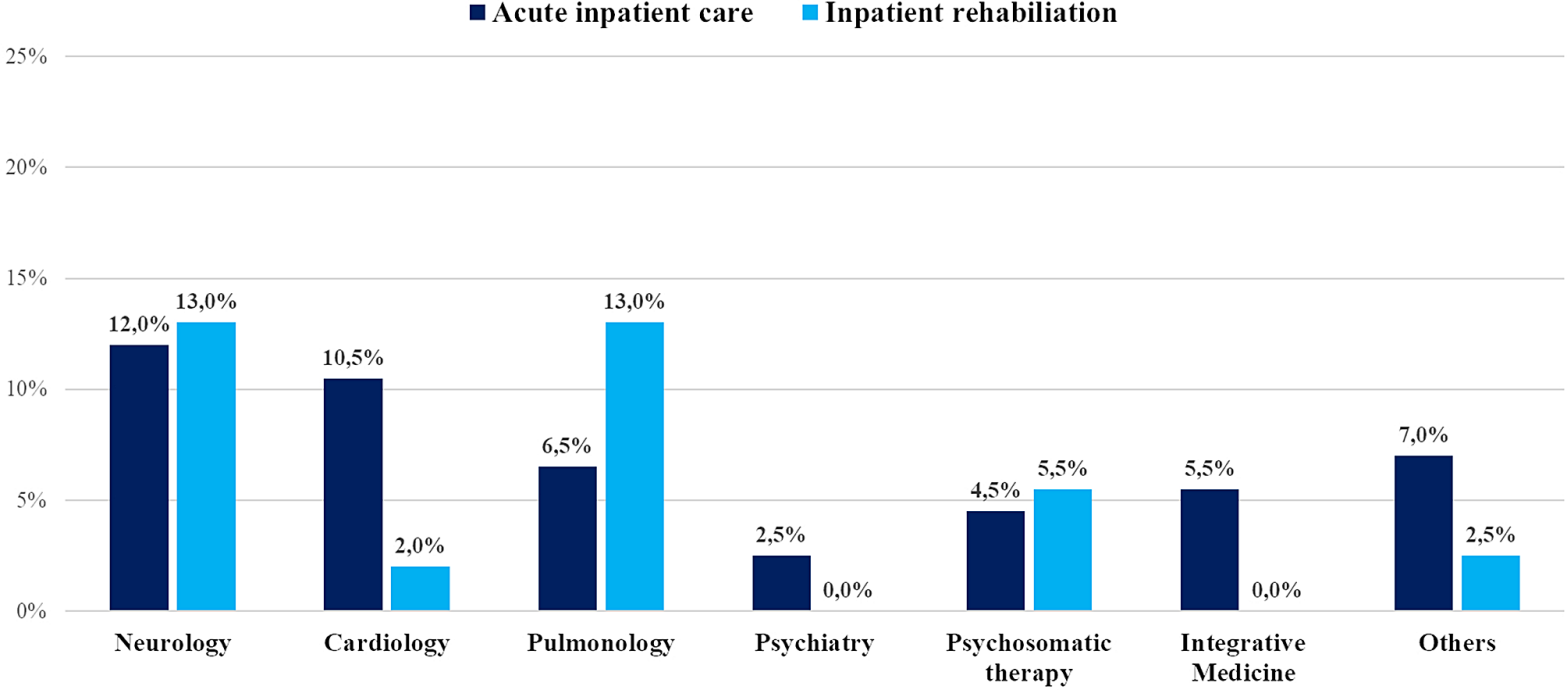
Distribution of inpatient admissions across specialties for acute and rehabilitative care (*N* = 200)

Among our patient cohort, 35.5% (*n* = 71) required admission to acute care hospitals, while 33.0% (*n* = 66) underwent inpatient rehabilitation at least once. The distribution across specialties is illustrated in Fig. 1. Cardiology and neurology emerged as primary focuses in acute care hospitalizations, accounting for 12.0% (*n* = 24) and 10.5% (*n* = 21) of cases, respectively. In terms of rehabilitation, neurology and pulmonology were equally prominent, each representing 13.0% (*n* = 26) of rehabilitation stays. Psychosomatic rehabilitation was recieved by 5.5% (n = 11) of patients.

### Outpatient referrals and treatments

In managing post-COVID syndrome, our patients predominantly sought expertise from cardiologists (76.5%, *n* = 153), pulmonologists (67.5%, *n* = 135), and neurologists (65.5%, *n* = 131), reflecting the diverse and complex symptomatology of post-COVID syndrome. Furthermore, 31.0% (*n* = 62) of our patients pursued outpatient psychotherapy to alleviate the psychological impacts of their long-term symptoms. A comprehensive overview of the range and frequency of further outpatient referrals and treatments pursued is detailed in Table 4.

**Table 4:** Outpatient referrals and treatments in the baseline sample according to medical records

Variables	Total sample (*N* = 200)
**Consulted medical specialist, n (%)** ^*^	
Cardiologist	153 (76.5)
Pulmonologist	135 (67.5)
Neurologist	131 (65.5)
ENT physician	39 (19.5)
Psychiatrist	38 (19.0)
Rheumatologist	25 (12.5)
Gastroenterologist	22 (11.0)
Orthopedist	13 (6.5)
Ophthalmologist	12 (6.0)
Endocrinologist	10 (5.0)
Osteopath	5 (2.5)
Dermatologist	4 (2.0)
Allergologist	4 (2.0)
Others	20 (10.0)
**Ambulatory rehabilitative therapies, n (%)** ^*^	
Physiotherapy	43 (21.5)
Ergotherapy	23 (11.5)
Respiratory therapy	9 (4.5)
Speech therapy	5 (2.5)
Specialized pain therapy	4 (2.0)
Others	5 (2.5)
**Further outpatient treatments, n (%)** ^*^	
Newly commenced outpatient psychotherapy	62 (31.0)
Presentation at another long- or post-COVID outpatient center	12 (6.0)
Others	9 (4.5)

^*^A simultaneous occurrence of multiple treatments options was possible

### Instrumental procedures

Apparative procedures were employed in a minority of patients within our sample. 3.5% (*n* = 7) underwent plasma exchange therapy (H.E.L.P. apheresis, immunoadsorption) to purify blood plasma from specific antibodies or reduce elevated lipid fractions. Hyperbaric oxygen therapy was administered to 1.5% (*n* = 3) of patients, while IHHT was utilized in 2.5% (*n* = 5).

Thermal procedures were also implemented in a subset of patients, with 3.5% (*n* = 7) undergoing such treatments. Among these, 1.5% (*n* = 3) received hyperthermia treatment, while 2.0% (*n* = 4) underwent cryotherapy.

### Subjectively perceived effectiveness of the therapeutic interventions

**Fig. 2:**
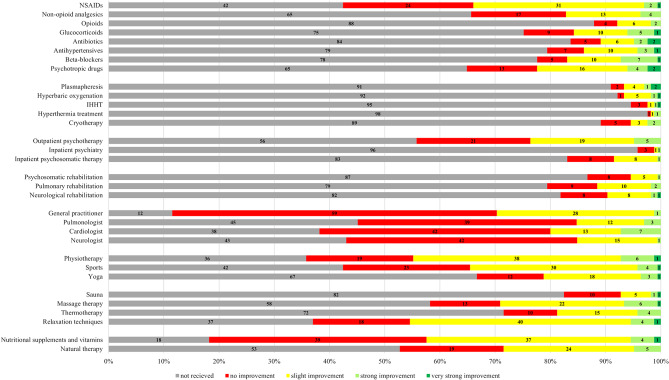
Self-reported outcome of the different therapy modalities as part of the follow-up questioning (N = 165)

The follow-up survey achieved a response rate of 82.5% (*n* = 165), with participants completing the questionnaire an average of 6.3 months (SD = 2.2) after their initial consultation at the post-COVID center. Among the treatments reported, patients most frequently used vitamins and nutritional supplements (81.8%, *n* = 135) as well as NSAIDs (57.6%, *n* = 95) to alleviate their post-COVID symptoms. Specifically, vitamin B and D, magnesium, and ibuprofen were the primary choices for symptom management. Physiotherapy and outpatient psychotherapy emerged as the most utilized treatments, with 64.2% (*n* = 106) and 44.2% (*n* = 73) of patients, respectively. The assessment of symptom improvement across various therapy modalities among patients is represented in Fig. 2.

By combining patient responses of ‘very strong improvement’ and ‘strong improvement,’ betablockers emerged as the most effective pharmacological approach for symptom control in post-COVID syndrome. A total of 31.6% (*n* = 12) of patients reported strong to very strong improvement under betablockers (Table 5), followed closely by antibiotics at 29.6% (*n* = 8), and corticosteroids at 24.4% (*n* = 10). Additionally, instrumental procedures demonstrated notable success in symptom improvement, with, for instance, 33.3% (*n* = 5) reporting significant alleviation of symptoms after undergoing plasmapheresis. Psychosomatic inpatient therapy, psychosomatic rehabilitation and outpatient psychotherapy were perceived as successful for strong or very strong symptom improvement only by a very small proportion of the sample (3.6%, *n* = 1; 4.5%, *n* = 1 and 11.0%, *n* = 8), respectively (Table 5).

**Table 5:** Proportion of therapy users with perceived strong to very strong symptom improvement

Variables	Number of therapy use (*n*)	Proportion of (very) strong symptom improvement among therapy users
**Medication groups, n (%)**		
NSAIDs^1^	95	5 (5.3)
Non-opioid analgesics	57	6 (10.5)
Opioids	20	3 (15.0)
Glucocorticoids	41	10 (24.4)
Antibiotics	27	8 (29.6)
Antihypertensives	34	7 (20.6)
Beta-blockers	38	12 (31.6)
Psychotropic drugs	58	10 (17.2)
**Instrumental procedures, n (%)**		
Plasmapheresis	15	5 (33.3)
Hyperbaric oxygenation	13	3 (23.1)
IHHT^2^	9	2 (22.2)
Hyperthermia treatment	4	1 (25.0)
Cryotherapy	18	4 (22.2)
**Psychological treatment, n (%)**		
Outpatient psychotherapy	73	8 (11.0)
Psychiatric inpatient therapy	7	1 (14.3)
Psychosomatic inpatient therapy	28	1 (3.6)
**Rehabilitation, n (%)**		
Psychosomatic rehabilitation	22	1 (4.5)
Pulmonary rehabilitation	34	3 (8.8)
Neurological rehabilitation	30	3 (10.0)
**Outpatient specialist, n (%)**		
General practitioner	146	2 (1.4)
Pulmonologist	90	5 (5.6)
Cardiologist	102	12 (11.8)
Neurologist	94	1 (1.1)
**Therapeutic movement, n (%)**		
Physiotherapy	106	12 (11.3)
Sports	95	7 (7.4)
Yoga	55	6 (10.9)
**Therapeutic relaxation, n (%)**		
Sauna	29	3 (10.3)
Massage therapy	69	11 (15.9)
Thermotherapy	47	7 (14.9)
Relaxation techniques	104	9 (8.7)
**Supplementary therapies, n (%)**		
Vitamins & nutritional supplements	135	9 (6.7)
Natural therapy	78	8 (10.3)

^1^Nonsteroidal anti-inflammatory drug (NSAIDs)

^2^Intermittent hypoxic-hyperoxic treatment (IHHT)

## Discussion

In the present study we analyzed the frequency of utilization and patient-reported effectiveness of various symptomatic therapies for post-COVID syndrome in a multidisciplinary specialized center. Post-COVID syndrome encompasses a diverse range of symptoms, including fatigue, cognitive impairments, respiratory issues, and cardiovascular problems, which significantly impair patients’ quality of life [4–6]. Recent literature highlights the complex pathogenesis contributing to post-COVID syndrome, which leads to this wide range of symptoms [4, 11].

It is becoming increasingly apparent that a variety of therapeutic methods, from cognitive-behavioral therapy [33] and rehabilitation programs [35] to medications like beta blockers [19], can provide symptom relief. While different symptom-oriented treatments are already used in general practice, there is a need to systematically assess their effectiveness to refine and enhance treatment regimens. To the best of our knowledge, this is the first study to evaluate the current utilization patterns of symptomatic therapies and compare their impact on patient-reported symptom improvement.

Our patient cohort primarily consisted of females aged between 40 and 49 years with tertiary education, a demographic pattern consistent with post-COVID patient statistics [42–44] and analogous to findings from other studies on treatment options for post-COVID [17, 18]. The low rate of hospital admissions during the acute infection phase observed in our cohort also aligns with data of other studies [34, 45].

Notably, 32.0% of our patients were diagnosed with chronic fatigue syndrome (CFS), a figure that aligns with data from other post-COVID studies [46]. Additionally, 6.0% of our patients exhibited symptoms of postural orthostatic tachycardia syndrome (POTS) following COVID-19, which is markedly higher than the estimated nationwide prevalence of 0.2% in Germany [47] and is consistent with findings from other research in this field [48].

Regarding pre-existing conditions, the prevalence of pre-existing depression in our sample was 21.5%, exceeding the lifetime prevalence of diagnosed depression of 11.6% reported for the general population in Germany [49]. Similarly, the prevalence of bronchial asthma was elevated at 17.5%, compared to the 12-month prevalence of 7.1% for women and 5.4% for men in Germany [50]. These findings align with other studies discussing bronchial asthma [45, 51] and depression [52, 53] as potential risk factors for the development and severity of post-COVID syndrome.

In this study, we assessed over 15 different therapeutic modalities for the symptomatic treatment of post-COVID syndrome in our study. Pharmacological interventions, particularly analgesics and psychotropic medications, have emerged as the cornerstone of post-COVID management, addressing a wide range of symptoms including fatigue and overall well-being [14]. In contrast, the utilization of plasmapheresis remains limited, primarily due to absence of definitive evidence-based guidelines for post-COVID syndrome and the substantial financial burden it imposes on patients [30, 54, 55]. While patients reports indicate promising outcomes in symptom alleviation with plasmapheresis, its limited application of 9.1% in our follow-up cohort precludes definitive conclusions about its overall effectiveness. Ongoing randomized controlled trials are underway to investigate its potential impact on post-COVID symptoms [45].

Our study found that neurological symptoms were the leading cause of inpatient admissions, accounting for 12.0% of acute care admissions and 13.0% of rehabilitation stays. This highlights the significant burden of neurological issues such as fatigue, brain fog and cognitive impairments associated with post-COVID syndrome [46]. Acute inpatient care additionally prioritizes cardiological evaluation and treatment, whereas in a rehabilitative context, the emphasis shifts towards pulmonology targeting post-COVID symptoms such as dyspnea and persistent cough [4, 8]. This prioritization is also reflected in our sample concerning outpatient care, where patients, in addition to their primary care physicians, primarily seek out cardiologists, pulmonologists, and neurologists to treat their post-COVID symptoms.

Although vitamins and dietary supplements are widely used due to their easy accessibility, their effectiveness appears so be limited. Outpatient psychotherapy, while frequently utilized, also yielded relatively modest symptom improvement. Psychosomatic inpatient therapy and psychosomatic rehabilitation exhibited similar results. This approach often emphasizes coping strategies and quality of life improvements rather than direct symptom alleviation in post-COVID syndrome [56, 57]. The relatively small proportion of patients who reported strong to very strong symptom improvement due to psychotherapeutic treatment may be explained by lacking psychosomatic treatment concepts at the time of data collection. In the meantime, first results demonstrated the effectiveness of psychosomatic rehabilitation in post-COVID patients, in particular concerning the reduction of depressive symptoms [58]. Thus, psychotherapeutic concepts have to be optimized.

The field of therapeutic approaches for post-COVID symptoms is broad and the optimal management strategy remains uncertain. However, findings from our study indicate that patients primarily contend with cardiological, neurological and pulmonary symptoms. In these areas, both outpatient and inpatient settings have seen the highest utilization of treatment options. The widespread use of analgesics also highlights the significant pain burden associated with post-COVID syndrome [59].

The observed diversity in treatment strategies underscores the heterogeneity of post-COVID symptomatology, hinting at potential subtypes within the syndrome [60, 61]. Alongside the quest for causal therapy, clear differentiation of these subtypes is essential to tailor symptomatic treatment to corresponding symptom clusters and patient needs, ensuring optimal care.

### Strengths and limitations

This study has several strengths. A strength of our study lies in the thorough review of medical records, including rehabilitation reports, documented consultations, and medical evaluations to capture the frequency of therapy application accurately. This approach allowed for a broad and realistic depiction of established treatments, without solely relying on patient self-reports, which may be subject to recall bias over time since illness onset. Another strength is the fusion of retrospective analysis of medical records with prospective data collection via an online follow-up survey, enabling patients’ personal assessment of therapy utilization and subjective evaluations of improvement. The large sample size is another relevant strength worth noting.

Our study also has some limitations. Despite our efforts to comprehensively capture all therapies, our retrospective assessment solely accounts for therapies documented through patient reports or available medical records, potentially overlooking non-reported or undocumented therapies. Consequently, the frequency of non-prescription or patient-initiated therapies may be underestimated. Additionally, an inherent selection bias exists due to tertiary referral to our outpatient center. Furthermore, the absence of a validated scale to assess patients’ subjective improvement may introduce variability in how progress is perceived and reported, possibly affecting the consistency of outcomes. Lastly, the absence of a control group from the broader population represents a further limitation.

We advocate for further research to assess the establishment and effectiveness of symptomatic therapies for post-COVID syndrome, utilizing longitudinal studies with larger sample sizes and control groups selected from the general population. Such investigations have the potential to deepen our comprehension of symptomatic treatment effectiveness and outcomes among post-COVID patients.

## Conclusion

This study presents initial insights into the utilization frequency and perceived effectiveness of symptomatic therapies in post-COVID syndrome. While our study suggests symptom amelioration with beta-blockers, glucocorticoids, and antibiotics, it highlights the absence of a universal therapy beneficial for all patients. Additionally, the moderate symptom improvement reported for psychotherapeutic treatments indicates the need for ongoing optimization of corresponding treatment concepts. Variations in therapeutic responses, contingent upon the predominant symptoms of post-COVID patients, advocate for a personalized treatment paradigm.

To improve treatment outcome, future research should aim to delineate distinct symptom clusters and explore tailored treatment modalities through randomized controlled trials with consideration of psychotherapeutic treatment concepts. Given the heterogeneity of symptoms, interdisciplinary collaboration is of utmost importance for a comprehensive understanding and effective management of post-COVID syndrome.

## Electronic supplementary material

Below is the link to the electronic supplementary material.

Supplementary Material 1

## Data Availability

This study involves a patient sample that will be surveyed prospectively at further measurement points. For this reason, a strict data protection concept has been stipulated, which does not allow the data to be made available for external access, even in anonymized form, in compliance with the European General Data Protection Regulation (GDPR).
