# Supplementary material for: Utilization frequency and patient-reported effectiveness of symptomatic therapies in post-COVID syndrome
Source: BMC Public Health. 2024 Sep 23;24:2577. doi: 10.1186/s12889-024-19951-3 (PMC11421202; doi:10.1186/s12889-024-19951-3)
Supplement: Supplementary file 1 — Supplementary Material 1 [file 12889_2024_19951_MOESM1_ESM.docx]

**Supplement 1**. Follow-up survey on the patient-perceived effectiveness of therapy modalities

Have you used any of the following therapeutic methods **to address your post-COVID symptoms** (**persistent symptoms lasting over 12 weeks after initial SARS-CoV-2 infection without alternative explanation**), and have you experienced symptom improvement as a result? (Please specify the treatments you've used and indicate if they've alleviated your post-COVID symptoms. Multiple responses are possible).

|  | Utilized | No improvement | Slight improvement | Strong improvement | Very strong improvment |
| --- | --- | --- | --- | --- | --- |
| **Medication** | | | | | |
| **NSAIDs** (e.g., Diclofenac, Ibuprofen, Acetylsalicylic Acid/Aspirin) |  |  |  |  |  |
| Name of the NSAID: ____________________ | | | | | |
| **Non-Opioids** (e.g., Metamizole) |  |  |  |  |  |
| Name of the Non-opioid: ____________________ | | | | | |
| **Opioids** (e.g., Tramadol, Tilidine, Morphine, Oxycodone, Codeine) |  |  |  |  |  |
| Name of the opioid: ____________________ | | | | | |
| **Corticosteroids** |  |  |  |  |  |
| Name of the corticosteroid: | | | | | |
| **Antibiotics** |  |  |  |  |  |
| Name of the antibiotic: ____________________ | | | | | |
| **Psychotropic drugs** |  |  |  |  |  |
| Name of the psychotropic drug: ____________________ | | | | | |
| **Cardiac medications** (e.g. Beta-blockers) |  |  |  |  |  |
| Name of the cardiac medication: ____________________ | | | | | |
| **Antihypertensives** |  |  |  |  |  |
| Name of the antihypertensive drug: ____________________ | | | | | |
| **Vitamins and nutritional supplement** (e.g. curcuma) |  |  |  |  |  |
| Name of the vitamin or nutritional supplement: | | | | | |
| **Other medications** |  |  |  |  |  |
| Name of the other medication: ____________________ | | | | | |
| **Instrumental procedures** | | | | | |
| Plasma exchange (plasmapheresis/separation) |  |  |  |  |  |
| Immunoadsorption (immunapheresis) |  |  |  |  |  |
| HELP-Apheresis |  |  |  |  |  |
| Hyperbaric oxygen therapy |  |  |  |  |  |
| Low Magnitude Mechanical Stimulation |  |  |  |  |  |
| Others: ____________________ | | | | | |
| **Psychotherapeutic/psychiatric/psychosomatic treatment** | | | | | |
| Outpatient psychotherapy |  |  |  |  |  |
| Inpatient psychiatric therapy |  |  |  |  |  |
| Inpatient psychosomatic therapy |  |  |  |  |  |
| Others: ____________________ | | | | | |
| **Outpatient specialists** | | | | | |
| General practitioner |  |  |  |  |  |
| Pulmonolgist |  |  |  |  |  |
| Cardiologist |  |  |  |  |  |
| Neurologist |  |  |  |  |  |
| Others: ____________________ | | | | | |
| **Inpatient rehabilitation** | | | | | |
| Psychosomatic rehabilitation |  |  |  |  |  |
| Pulmonary rehabilitation |  |  |  |  |  |
| Neurological rehabilitation |  |  |  |  |  |
| Others: ____________________ | | | | | |
| **Therapeutic movement** | | | | | |
| Physiotherapy |  |  |  |  |  |
| Sports |  |  |  |  |  |
| Yoga |  |  |  |  |  |
| Others: ____________________ | | | | | |
| **Therapeutic relaxation** | | | | | |
| Sauna |  |  |  |  |  |
| Massage therapy |  |  |  |  |  |
| Warmth or thermotherapy |  |  |  |  |  |
| Relaxation techniques |  |  |  |  |  |
| Others: ____________________ | | | | | |
| **Complementary medicine** | | | | | |
| Vitamins and nutritional supplements |  |  |  |  |  |
| Natural therapy |  |  |  |  |  |
| Others: ____________________ | | | | | |
